# ISSLS PRIZE IN BIOENGINEERING SCIENCE 2019: biomechanical changes in dynamic sagittal balance and lower limb compensatory strategies following realignment surgery in adult spinal deformity patients

**DOI:** 10.1007/s00586-019-05925-2

**Published:** 2019-03-02

**Authors:** Jeannie F. Bailey, Robert P. Matthew, Sarah Seko, Patrick Curran, Leslie Chu, Sigurd H. Berven, Vedat Deviren, Shane Burch, Jeffrey C. Lotz

**Affiliations:** 1 Department of Orthopaedic Surgery, University of California, San Francisco, USA; 2 Department of Electrical Engineering and Computer Science, University of California, Berkeley, USA

**Keywords:** Sagittal balance, Spinal biomechanics, Adult spinal deformity, Compensatory mechanisms, Sit-to-stand, Proximal junctional kyphosis, Post-surgical outcomes

## Abstract

**Study design:**

A longitudinal cohort study.

**Objective:**

To define a set of objective biomechanical metrics that are representative of adult spinal deformity (ASD) post-surgical outcomes and that may forecast post-surgical mechanical complications.

**Summary of background data:**

Current outcomes for ASD surgical planning and post-surgical assessment are limited to static radiographic alignment and patient-reported questionnaires. Little is known about the compensatory biomechanical strategies for stabilizing sagittal balance during functional movements in ASD patients.

**Methods:**

We collected in-clinic motion data from 15 ASD patients and 10 controls during an unassisted sit-to-stand (STS) functional maneuver. Joint motions were measured using noninvasive 3D depth mapping sensor technology. Mathematical methods were used to attain high-fidelity joint-position tracking for biomechanical modeling. This approach provided reliable measurements for biomechanical behaviors at the spine, hip, and knee. These included peak sagittal vertical axis (SVA) over the course of the STS, as well as forces and muscular moments at various joints. We compared changes in dynamic sagittal balance (DSB) metrics between pre- and post-surgery and then separately compared pre- and post-surgical data to controls.

**Results:**

Standard radiographic and patient-reported outcomes significantly improved following realignment surgery. From the DSB biomechanical metrics, peak SVA and biomechanical loads and muscular forces on the lower lumbar spine significantly reduced following surgery (− 19 to − 30%, all *p* < 0.05). In addition, as SVA improved, hip moments decreased (− 28 to − 65%, all *p* < 0.05) and knee moments increased (+ 7 to + 28%, *p* < 0.05), indicating changes in lower limb compensatory strategies. After surgery, DSB data approached values from the controls, with some post-surgical metrics becoming statistically equivalent to controls.

**Conclusions:**

Longitudinal changes in DSB following successful multi-level spinal realignment indicate reduced forces on the lower lumbar spine along with altered lower limb dynamics matching that of controls. Inadequate improvement in DSB may indicate increased risk of post-surgical mechanical failure.

**Graphical abstract:**

These slides can be retrieved under Electronic Supplementary Material.
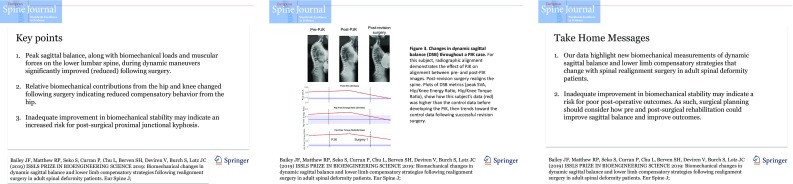

**Electronic supplementary material:**

The online version of this article (10.1007/s00586-019-05925-2) contains supplementary material, which is available to authorized users.

## Introduction

Adult spinal deformity (ASD) is an increasingly prevalent and costly problem [1], often requiring a lifetime of medical treatment, including surgical intervention. Surgical correction of ASD seeks to restore sagittal balance, which refers to the ability to maintain a mechanically effective center of pressure (gravity line) [2] via postural control of the spine and lower extremities while upright, including both quiet standing and more dynamic motions of daily life. Prior pivotal work showed an association between patient-reported health status and sagittal imbalance based on a measure of the sagittal vertical axis (SVA) from standing radiography [3]. These associations are drawn from questionnaires for patient-reported outcomes (PROs) for pain, disability, and quality of life, plus quantitative measurements of spinal alignment in standing radiography. However, questionnaires are subjective, and assessing spinal alignment from static standing radiography as a proxy for sagittal balance does not consider the effect of postural control and lower extremity mechanics that contribute to sagittal balance during dynamic motions of daily living.

Static SVA does not completely capture a patient’s ability to actively stabilize their spine when performing dynamic movements. For example, gait analyses of sagittal balance reveal compensatory mechanisms recruited from the lower extremities [4, 5]. In addition, the effect of muscular fatigue from short walks can significantly worsen standing SVA [6], which indicates that in-clinic radiographs that inform surgical realignment may not accurately reflect the patient’s condition outside the clinic. Consequently, assessing postural stability during dynamic tasks may pave the way for a better understanding of postural compensatory mechanisms, the hip–spine complex, and risk of post-surgical mechanical complications, including proximal junctional kyphosis (PJK) and failure [5,7]. The incidence of PJK is reported to occur following 20–39% [8] of cases and often requires a subsequent revision surgery, creating an added burden on the patient, clinicians, and hospitals.

Routine clinical assessment of dynamic sagittal balance is impractical given the setup time, space requirements, and high technology cost. Simple dynamic functional tasks, like a sit-to-stand (STS) maneuver, do not require the same amount of space as gait analysis and are diagnostically appropriate given that these tasks are commonly arduous for ASD patients due to the necessary whole body balance and postural control. Using 3D depth mapping sensor technology, we have developed an efficient means for collecting in-clinic motion analysis data. Integrated mathematical methods for noise filtering and kinematic constraints provide high-fidelity joint-position tracking for biomechanical modeling, enabling reliable dynamic quantification of spine, hip, and knee biomechanics. We used this novel technology to longitudinally assess DSB from a cohort of 15 ASD patients undergoing multi-level spinal fusion, and to compare pre- and postoperative DSB data to 10 healthy controls. The purpose of this study was to define a set of objective biomechanical metrics that are representative of post-surgical outcomes and may, in the future, be used to predict risk of post-surgical PJK.

## Materials and methods

### Sample

With IRB approval, we collected in-clinic motion analysis and outcomes data from ASD patients during routine pre- and post-surgical clinical visits at the University of California, San Francisco. This study includes 15 adult patient subjects and 10 healthy control subjects that were able to perform an unassisted STS maneuver.

### In-clinic motion analysis and biomechanical modeling

**Fig. 1:**
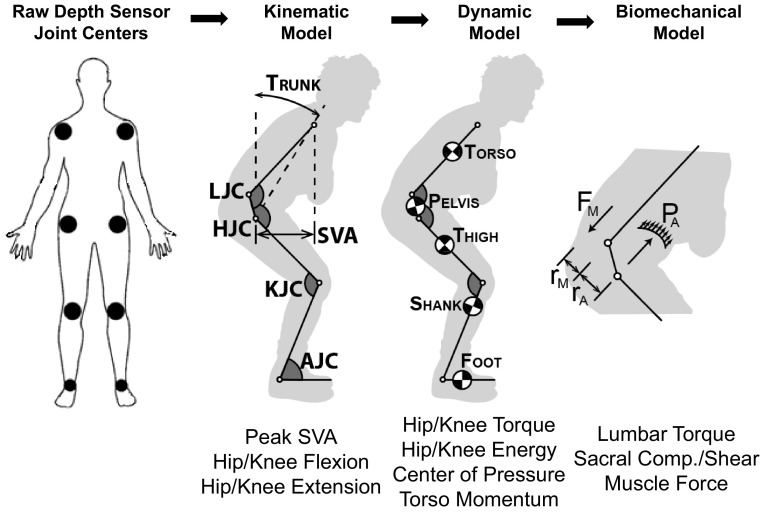
Processing pipeline for the proposed depth camera system for recovering STS data. Raw estimates of joint center position are sequentially processed to obtain kinematic, dynamic, and biomechanical metrics

Patients were asked to complete as many unassisted STS maneuvers as they could during a maximum of nine maneuvers (three separate trials of three maneuvers each). An RGB-depth camera (Kinect 2, Microsoft, Inc.) tracked 3D joint positions from the frontal view during the entire STS maneuvers. Raw estimates of joint location were filtered using an unscented Kalman filter (UKF) [9] and an allometrically scaled, patient-specific rigid body model [10]. The computed kinematic, kinetic, and dynamic parameters were then used to estimate lumbar loading using a sagittal plane model of intra-abdominal pressure and the spine extensors (Fig. 1) [11]. The kinematic, kinetic, and dynamic metrics obtained from this depth camera system have been validated for the sit-to-stand action [12, 13].

### Dynamic sagittal balance metrics

We calculated a series of DSB parameters from the in-clinic motion data of the unassisted STS functional maneuver (Table 1). While the patient rose from the seated position, we quantified: time (s), peak SVA, horizontal and vertical momentum (1/s), joint torque (1/s^2^), peak compressive and shear force on sacrum from the dynamics of the trunk (1/s), peak spinal extensor muscle force (1/s^2^), peak flexion and extension of the hip and knee (°), total hip and knee motoric energy (1/s^2^), max sagittal offset of CoP during dynamic maneuver and sagittal offset in quiet standing posture. All metrics, except time and flexion/extension, were normalized based on subject height and mass—for this reason, SVA and COP did not have units.

**Table 1:** Dynamic sagittal balance metrics

Variable name	Variable definition	Scaling variable	Units
Time	Time between quiet sitting and quiet standing. Quiet sit and stand are the last and first time points where the subject is in a seated or standing position and the energy of the torso is effectively zero	None	s
Peak SVA	Peak sagittal distance between the hip and shoulder centers	Height	None
Horizontal and vertical momentum	The horizontal and vertical momenta of the torso as seen in the world frame	Height*Mass	1/s
Torque at the lower lumbar spine, hip and knee	Peak estimated torque at the L5/S1, hip, and knee joints	Height^2^*Mass	1/s^2^
Sacral compression and shear	Peak compressive and shear forces at S1 based on the musculoskeletal model from Chaffin et al. (2006)	Height*Mass	1/s^2^
Spinal extensor muscle force	Peak contractile force on the back extensor muscles based on musculoskeletal model from Chaffin et al. (2006)	Height*Mass	1/s^2^
Flexion and extension of the hip and knee	Maximum flexion and extension angles of hip and knee joints	None	°
Total motoric energy of the hip and knee	Integral of the instantaneous power by time over the whole STS action	Height^2^*Mass	1/s^2^
Dynamic CoP	Maximum sagittal distance from the center of the foot to the CoP	Subject foot length (from allometric scaling)	None
Standing CoP	Distance from the center of the foot to the CoP during quiet standing	Subject foot length (from allometric scaling)	None

### Patient outcomes

Beyond data collected from motion analysis, we collected radiographic SVA and patient-reported outcomes (PROs) for Oswestry Disability Index for low back disability (ODI, 0–100), VAS for back pain (0–10), and EQ5D utility for health-related quality of life (0–1) when attained during the same clinical visit.

### Statistical analyses

DSB data used for statistical analysis were averaged over multiple STS trials per subject. Pre- to post-surgical changes were compared using paired *t* tests. Pre- and post-surgical data were separately compared to control data using unpaired *t* tests. Significance was based on *p* < 0.05. All statistical analyses were done using Stata 15.1 (Stata Corp, College Station, TX, USA).

## Results

### Pre- to post-surgical changes in dynamic sagittal balance and outcomes

From the 15 ASD patients (age 62 ± 10 years; females, *n* = 13; males, *n* = 2) for which we have complete post-surgical follow-up data, we compared changes in DSB before and after realignment surgery. Patient-reported outcomes all significantly improved on average following surgery, including 25% decrease in ODI (52–38, *p* = 0.03), 27% decrease in VAS (6.6–4.8, *p* = 0.05), and 34% increase in EQ5D utility (0.50–0.67, *p* = 0.03). In addition, radiographic SVA significantly improved by an average of 53% (96.2–45.6 mm, *p* < 0.001).

Many DSB measurements significantly changed following surgery (Tables 2 and 3). Peak SVA decreased (− 28%, *p* < 0.001), and time needed to rise to a stable standing position decreased (− 37%, *p* < 0.001). Biomechanical forces on the spine changed, including reduced torque on the lower lumbar spine (− 29%, *p* < 0.01) and, more specifically, reduced compressive and shear forces acting on the sacrum from altered dynamics of the trunk (max compression: − 23%, *p* = 0.02; max shear: − 19%; *p* = 0.03). In addition, peak spinal extensor muscle force decreased as well (− 30%, *p* < 0.01). Flexion at the hip was lower (− 28%, *p* < 0.001), as was the peak torque (− 33%, *p* < 0.001) and energy (− 65%, *p* < 0.01) required from the hip to complete the maneuver. Flexion at the knee was greater (+ 7%, *p* = 0.04), as was energy (+ 26%, *p* = 0.04).

**Table 2:** Dynamic sagittal balance metrics for controls and patient subjects at pre-surgery and post-surgery time points

	Controls		Pre-surgery		Post-surgery	
	Mean ± SD	95% CI	Mean ± SD	95% CI	Mean ± SD	95% CI
Time	1.5 ± 0.1	1.5, 1.6	3.0 ± 1.0	2.5, 3.6	1.9 ± 0.5	1.7, 2.2
Peak SVA	142 ± 19.9	127.8, 156.2	229.3 ± 34.9	210.0, 248.7	164.0 ± 39.4	142.2, 185.8
Vertical momentum	0.22 ± 0.02	0.21, 0.23	0.18 ± 0.09	0.14, 0.23	0.17 ± 0.04	0.15, 0.20
Horizontal momentum	0.15 ± 0.02	0.14, 0.17	0.14 ± 0.09	0.09, 0.19	0.13 ± 0.03	0.11, 0.14
Standing COP	0.09 ± 0.48	− 0.25, 0.43	0.37 ± 0.48	0.10, 0.63	0.17 ± 0.56	− 0.15, 0.48
Dynamic COP	0.35 ± 0.25	0.18, 0.53	0.29 ± 0.44	0.05, 0.52	0.46 ± 0.33	0.28, 0.64
Lumbar torque	0.37 ± 0.70	0.31, 0.42	0.49 ± 0.20	0.48, 0.59	0.35 ± 0.08	0.31, 0.39
Spinal extensor muscle force	9.7 ± 1.5	8.7, 10.7	12.4 ± 4.7	9.8, 15.0	8.7 ± 1.9	7.6, 9.7
Max sacral compression	10.8 ± 1.2	9.9, 11.7	12.6 ± 4.4	10.2, 15.1	9.7 ± 1.5	8.9, 10.5
Max sacral shear	6.2 ± 0.6	5.7, 6.6	6.7 ± 2.5	5.4, 8.1	5.4 ± 0.8	5.0, 5.8
Hip torque	0.44 ± 0.10	0.38, 0.52	0.63 ± 0.23	0.51, 0.76	0.42 ± 0.10	0.37, 0.48
Hip energy	0.07 ± 0.04	0.05, 0.10	0.20 ± 0.13	0.13, 0.28	0.07 ± 0.03	0.05, 0.09
Max hip flexion	96.8 ± 4.4	93.7, 100.0	66.4 ± 13.5	58.9, 73.9	85.1 ± 10.8	79.1, 91.1
Max hip extension	176.8 ± 0.7	176.3, 177.3	167.0 ± 9.9	161.5, 172.4	169.4 ± 6.0	166.0, 172.7
Knee torque	1.3 ± 0.1	1.2, 1.4	1.22 ± 0.2	1.09, 1.34	1.33.0 ± 0.2	1.24, 1.42
Knee energy	0.46 ± 0.1	0.40, 0.51	0.55 ± 0.1	0.49, 0.61	0.69 ± 0.30	0.52, 0.85
Max knee flexion	89.8 ± 6.7	85.0, 94.6	83.4 ± 10.8	77.5, 89.4	77.7 ± 7.0	73.9, 81.6
Max knee extension	179.6 ± 2.3	177.9, 181.3	175.2 ± 12.7	168.6, 182.7	175.2 ± 8.1	171.0, 179.5

Data include mean, standard deviation, and 95% confidence interval. Units and definitions for DSB metrics can be found in Table 1

**Table 3:** Between-group comparisons in DSB metrics

	Pre-surgical compared to post-surgical (paired)	Pre-surgical compared to controls (unpaired)	Post-surgical compared to controls (unpaired)
Time	− 37% *p* < 0.001	+ 38%, *p* < 0.0001	+ 15%, *p* = 0.04
Peak SVA	− 28%, *p* < 0.001	+ 101%, *p* < 0.0001	+ 30%, *p* = 0.002
Vertical momentum	n.s.	n.s.	− 30%, *p* = 0.001
Horizontal momentum	n.s.	n.s.	− 15%, *p* = 0.01
Standing COP	n.s.	n.s.	n.s.
Dynamic COP	n.s.	n.s.	n.s.
Lumbar torque	− 29%, *p* = 0.002	+ 32%, 0.02	n.s.
Peak muscle force	− 30%, *p* = 0.002	+ 22%, *p* = 0.05	n.s.
Max sacral compression	− 23%, *p* = 0.007	n.s.	− 10%, *p* = 0.03
Max sacral shear	− 19%, *p* = 0.02	n.s.	− 15%, *p* = 0.007
Hip torque	− 33%, *p* = 0.0006	+ 30%; *p* = 0.01	n.s.
Hip motoric energy	− 65%, *p* = 0.005	+ 64%, *p* = 0.003	n.s.
Max hip flexion	− 28%, *p* < 0.001	− 31%, *p* < 0.0001	− 16%, *p* = 0.0001
Max hip extension	n.s.	− 4%, *p* < 0.001	− 6%, *p* < 0.01
Knee torque	n.s.	n.s.	n.s.
Knee motoric energy	+ 26%, *p* = 0.04	+ 16%, *p* = 0.01	+ 31%, *p* = 0.02
Max knee flexion	+ 7%, *p* = 0.04	n.s.	+ 13%, *p* < 0.001
Max knee extension	n.s.	n.s.	n.s.

Data between pre- and post-surgery time points were compared using a paired *t* test. Data between the separate surgery time points and controls were compared using an unpaired *t* test. Data were reported for significant results, and nonsignificant data (n.s., *p* > 0.05) were not reported

Variables that did not significantly change following surgical intervention include standing or dynamic center of pressure, vertical and horizontal momentum, hip and knee maximum extension (at standing), and knee torque.

### Comparing DSB between surgical patients and healthy controls

**Fig. 2:**
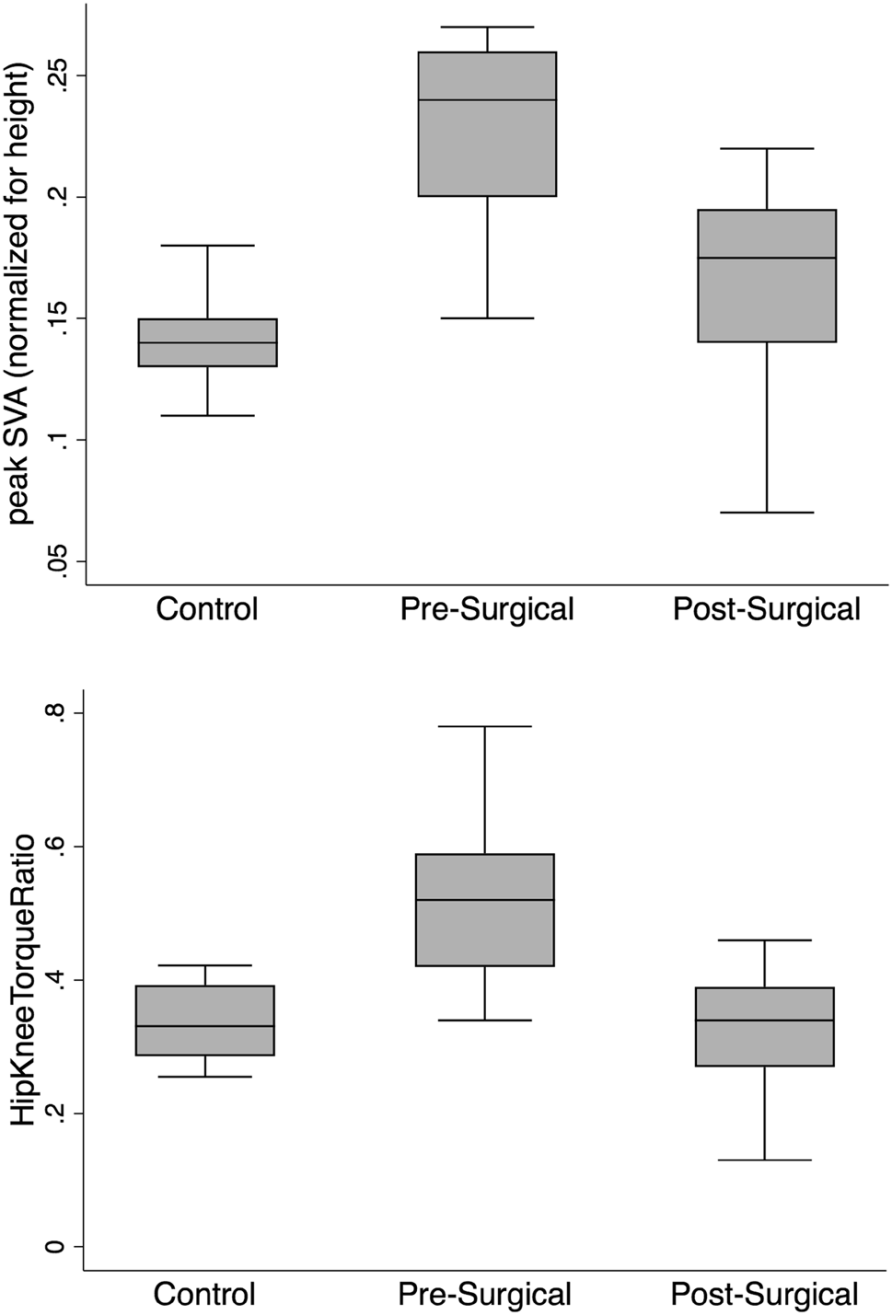
Box plots for group comparison in peak SVA and hip/knee torque ratio. Both peak SVA and hip/knee torque ratio significantly decrease between pre- and post-surgery time points. For peak SVA, post-surgical is 28% lower than pre-surgical (*p* < 0.001). For hip/knee torque ratio, post-surgical is 19% points lower than pre-surgical (*p* < 0.001). Both metrics were significantly higher for the pre-surgical data compared to the controls, but the post-surgical data did not differ from the controls, showing improvement for the surgical patients

We compared both pre- and post-surgical DSB data from the ASD patients to 10 healthy control subjects (age 31 ± 10; females, *n* = 3; males, *n* = 7). Comparing DSB between our pre-surgical patient data and healthy controls, we found significant differences in some DSB variables that also showed significant change with surgery (Tables 2 and 3). A number of the DSB variables were significantly different from the control data prior to surgery (time: + 38%; peak SVA: + 101% (Fig. 2); lumbar torque: + 32%; spinal extensor muscle force: + 22%; hip torque: + 30%; hip energy: + 64%; hip flexion: − 31%, knee energy: + 16%). Following surgery, some of these variables improved by closing the gap in differences with the controls (time: 15%; peak SVA: + 30% (Fig. 2); hip flexion: − 16%), and others improved by becoming not significantly different than the controls (lumbar torque; spinal extensor muscle force; hip torque; hip energy).

Interestingly, several pre-surgical variables that were not different from the control data were different at the post-surgical time point, including vertical and horizontal momentum (− 30%, − 15%), maximum compression and shear force on the sacrum from the dynamics of the trunk (− 10%, − 15%), and maximum knee flexion (+ 13%).

### Pre- to post-surgical changes in relative ratios of kinetic variables between hip and knee

Ratios for torque and energy between the hip and knee significantly reduced following surgery [torque: 52% to 33% (Fig. 2), *p* < 0.001; energy: 41% to 11%, *p* = 0.002]. Pre-surgical ratios were significantly higher than controls [torque: 52% to 34% (Fig. 2), *p* < 0.001; energy: 41% to 16%, *p* = 0.02], but post-surgical values did not significantly differ from controls.

### PJK case study

**Fig. 3:**
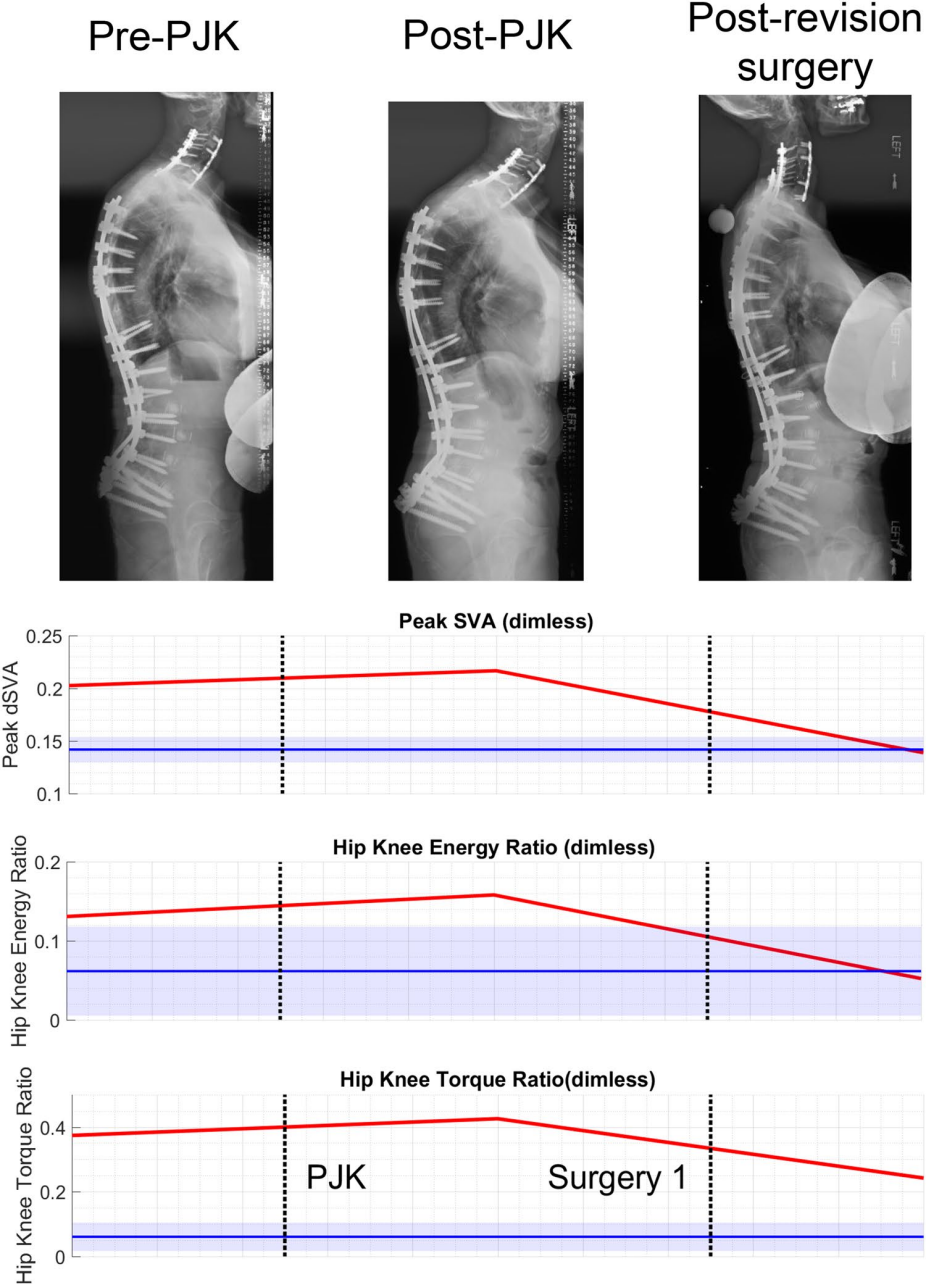
Changes in DSB throughout a PJK case. For this subject, radiographic alignment demonstrates the effect of PJK on alignment between pre- and post-PJK images. Post-revision surgery realigns the spine. Plots of DSB metrics (peak SVA, hip/knee energy ratio, hip/knee torque ratio) show how these subject’s data (red) were higher than the control data before developing the PJK and then trends toward the control data following successful revision surgery

One patient developed a PJK complication that required revision surgery. For this patient, we have data from three time points: (1) follow-up from prior surgery, (2) visit following the onset of PJK, and (3) follow-up visit after revision surgery. Standout DSB metrics as potential risk factors include peak SVA and relative ratios of hip and knee torque and energy. These values were higher than the controls prior to PJK, and following revision surgery, they more closely matched the control data (Fig. 3).

## Discussion

Our goal was to use a validated in-clinic functional assessment tool to identify biomechanical metrics that associate with post-surgical outcomes. Current measures for surgical planning include static radiographic alignment and patient-reported questionnaires for pain, disability, and health status. Our data highlight new biomechanical measurements of DSB and lower limb compensatory strategies that change with spinal realignment surgery in ASD patients: (1) reduced peak SVA and biomechanical loads and muscular forces on the lower lumbar spine, and (2) altered relative biomechanical contributions from the hip and knee indicating reduced compensatory behavior from the hip. Changes in pre- to post-surgical DSB data moved toward the values from the healthy controls with most of the post-surgical metrics becoming equivalent to controls.

Dynamic biomechanical assessment of sagittal balance and lower limb compensation may fill gaps between static radiographic spinal alignment and patient-reported health status. Sagittal imbalance is thought to encompass how spinal malalignment can cause unwanted skewness in one’s center of pressure. However, when considering compensatory alignment of the pelvis and lower limbs, a malaligned spine may not shift center of pressure [14], but, instead, initiate mechanically ineffective compensatory balance strategies in order to maintain the position of the center of pressure between the feet [15]. Specific results from our study show that during a dynamic task requiring balance and postural stability, ASD patients deviate from healthy controls with motions relating to higher torque on and muscle forces needed from the lower lumbar spine, more work from the hip and in turn less active work from the knees in order to maintain a stable position of the center of pressure. These metrics are shown to improve with spinal realignment surgery.

Prior studies demonstrate improvement in functional performance after spinal realignment surgery. A heightened effort to understand gait motion behavior in ASD patients has emerged [5], and one study has shown results supporting improved kinematic metrics in ASD patients at 1 year and 2 years following surgery [16]. Main findings include increased thoracopelvic, hip, and knee range of motion during the gait cycle. While these findings demonstrate improvement in gait after surgery, the ability to integrate these findings into clinical practice to support more effective outcomes remains a challenge. Although gait is the most studied dynamic functional and is often difficult for ASD patients, gait may be less practical for routine clinical assessment using motion analysis. Other dynamic functional tasks requiring active postural stability may be more challenging than gait for pre-surgical ASD patients due to heightened loading on the spine and lower limbs.

Our study utilized an STS maneuver for its practical implementation within a clinic room and requiring adequate postural stability to effectively rise from a stable seated position to a stable standing position. STS is often employed as a clinically meaningful functional task for spine patients. Other studies that have assessed STS on spine function primarily focus on its relevance as functional test for low back pain patients [17–22] and often include a different form of the test (e.g., 5-time STS [22, 23]). In studies that have assessed sagittal plane mechanics of STS in low back pain patients, most only distinguish degrees of motion at the trunk, hip, and knee and are limited with a cross-sectional study design. The few studies that have measured joint kinetics during STS in low back pain patients show linkages between compensatory lower limb biomechanics and relatively higher loads on the spine [17, 24]. Our present study uniquely examines STS in ASD patients whose lower limb compensatory biomechanics are likely a compensatory response to poor postural control from spinal malalignment; however, the lower limb compensatory biomechanics could be exacerbating poor outcomes by increasing loads on the spine.

**Fig. 4:**
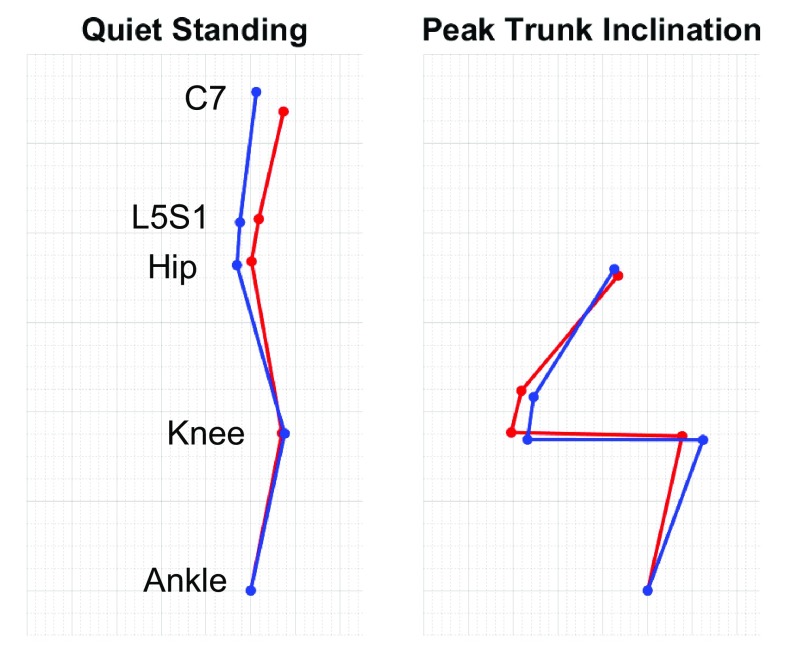
Biomechanical model of pre- to post-surgical changes for example subject. Sagittal profile of our biomechanical model of a patient subject demonstrates the changes in spine, hip, knee alignment following surgery (red is pre-surgery and blue is post-surgery). During quiet standing, subject demonstrates correction of alignment that would be indicated from static radiography. However, at peak trunk inclination during STS maneuver, subject is utilizing less of a leaning posture that is shown to place greater biomechanical force on the spine

We found that biomechanical standing strategies from the STS task improve following spinal realignment surgery. Our results support that pre-surgical ASD patients employ a ‘quasi-static leaning’ strategy to transition from a stable seated to stable standing position [25]. Using this leaning strategy, the subject leans as far forward as possible to move their torso directly above their feet, before engaging their knees and hips to rise. This essentially recruits postural compensatory mechanics to maintain a passively stable position of one’s center of pressure. Our results showing post-surgical improvements in dynamic peak SVA, lumbar torque, extensor muscle force, and hip torque and energy suggest that subjects become less reliant on the quasi-static lean strategy (Fig. 4). Furthermore, subjects that are exercising more strength from the knees following surgery may support potential benefits from leg strengthening exercises for improved outcomes.

Our results allude to potential predictors for PJK risk. The rates of revision surgery due to PJK/PJF following multi-level fusion in ASD patients are high, and a lack of understanding regarding risks persists. Some known risk factors for PJK that relate to possibly excessive loading on the spine include large changes in radiographic SVA [26], poor paraspinal muscle quality [27], and neuromuscular comorbidities [7]. ASD patients with ambitious realignments and overcorrection are also at a higher risk of post-surgical PJK/PJF [28]. One patient subject from our present study experienced a PJK complication requiring revision surgery, and their longitudinal DSB behaviors indicate that a lack of improvement in peak SVA and changes in relative contributions of torque and energy in the hip and knee may be risk factors for PJK (Fig. 3). A better understanding of how patient movement may place excessive loads on the spine would help develop pre- and post-surgical rehabilitation strategies to improve postoperative DSB and prevent overloading the vulnerable region between unfused and (newly) fused spinal segments.

Limitations for this current work include, firstly, our relatively small sample size. The purpose of this study was to establish how DSB changes following realignment surgery to create a number of clinically meaningful metrics for deciphering outcomes from DSB assessments. Future work will collect a larger sample to begin exploring associations between patient-reported outcome scores and radiographic alignment data with DSB metrics.

Other limitations regard possible constraints from the biomechanical modeling. One is how we normalized the DSB biomechanical metrics for mass and stature. It is important that we normalized data so that DSB metrics were comparable between subjects regardless of body size, but it creates values and units that can be difficult to understand. Another limitation from the modeling being the spine is represented as primarily one segment. Future work aims to improve the model to distinguish thoracic motion from lumbar. Lastly, age differences between our patient subjects and healthy controls could confound our analyses. However, one study found loads on the lower lumbar spine during STS to be similar between relatively younger and older individuals provided both were asymptomatic [29].

In conclusion, our study utilizes a novel in-clinic approach for measuring dynamic biomechanical metrics of sagittal balance through the spine, hip, and knee. This technology enabled us to track changes in sagittal balance biomechanics following spinal realignment surgery in ASD patients and introduce new objective outcomes. Longitudinal changes in dynamic sagittal balance following successful multi-level spinal realignment indicate changes in spinal biomechanics reducing forces acting on the lower lumbar spine along with changes in lower limb dynamics matching that of healthy controls. Furthermore, potential risk factors for PJK may include inadequate improvement in peak SVA and relative ratios of hip and knee torque and energy. Our results suggest that surgical planning should consider how pre- and post-surgical rehabilitation could improve sagittal balance and improve outcomes.

## Electronic supplementary material

Below is the link to the electronic supplementary material.

Supplementary material 1 (PPTX 1132 kb)

## References

[1] Ames CP, Scheer JK, Lafage V (2016). Adult spinal deformity: epidemiology, health impact, evaluation, and management. Spine Deform.

[2] Schwab F, Lafage V, Boyce R (2006). Gravity line analysis in adult volunteers: age-related correlation with spinal parameters, pelvic parameters, and foot position. Spine.

[3] Glassman SD, Bridwell K, Dimar JR (2005). The impact of positive sagittal balance in adult spinal deformity. Spine.

[4] Arima H, Yamato Y, Hasegawa T (2017). Discrepancy between standing posture and sagittal balance during walking in adult spinal deformity patients. Spine.

[5] Diebo BG, Shah NV, Pivec R (2018). From static spinal alignment to dynamic body balance. JBJS Rev.

[6] Bae J, Theologis AA, Jang J-S (2017). Impact of fatigue on maintenance of upright posture: dynamic assessment of sagittal spinal deformity parameters after walking 10 min. Spine.

[7] Glassman SD, Coseo MP, Carreon LY (2016). Sagittal balance is more than just alignment: why PJK remains an unresolved problem. Scoliosis Spinal Disord.

[8] Nguyen N-LM, Kong CY, Hart RA (2016). Proximal junctional kyphosis and failure-diagnosis, prevention, and treatment. Curr Rev Musculoskelet Med.

[9] Julier SJ, Uhlmann JK (1997) New extension of the Kalman filter to nonlinear systems. In: Kadar I (ed) Proc of SPIE, vol 3068, pp 182–193

[10] Dumas R, Cheze L, Verriest JP (2007). Adjustments to McConville et al. and Young et al. body segment inertial parameters. J Biomech.

[11] Zhao F, Pollintine P, Hole BD (2005). Discogenic origins of spinal instability. Spine.

[12] Matthew RP, Seko S, Bajcsy R et al (2018) Kinematic and kinetic validation of an improved depth camera motion assessment system using rigid bodies. IEEE J Biomed Health Inform [Epub ahead of print] 10.1109/JBHI.2018.2872834 30281504

[13] Matthew RP, Seko S, Bailey JF, Bajcsy R, Lotz J. (2019) Estimating sit-to-stand dynamics using a single depth camera. IEEE J Biomed Health Inform [Epub ahead of print] 10.1109/JBHI.2019.2897245 30716057

[14] Lafage V, Schwab F, Skalli W (2008). Standing balance and sagittal plane spinal deformity: analysis of spinopelvic and gravity line parameters. Spine.

[15] Brech GC, Alonso AC, Luna NMS, Greve JM (2013). Correlation of postural balance and knee muscle strength in the sit-to-stand test among women with and without postmenopausal osteoporosis. Osteoporos Int.

[16] Engsberg JR, Bridwell KH, Wagner JM (2003). Gait changes as the result of deformity reconstruction surgery in a group of adults with lumbar scoliosis. Spine.

[17] Shum GLK, Crosbie J, Lee RYW (2007). Three-dimensional kinetics of the lumbar spine and hips in low back pain patients during sit-to-stand and stand-to-sit. Spine.

[18] Fotoohabadi MR, Tully EA, Galea MP (2010). Kinematics of rising from a chair: image-based analysis of the sagittal hip-spine movement pattern in elderly people who are healthy. Phys Ther.

[19] Sanchez-Zuriaga D, Lopez-Pascual J, Garrido-Jaen D (2011). Reliability and validity of a new objective tool for low back pain functional assessment. Spine.

[20] Christe G, Redhead L, Legrand T (2016). Multi-segment analysis of spinal kinematics during sit-to-stand in patients with chronic low back pain. J Biomech.

[21] Hemming R, Sheeran L, van Deursen R, Sparkes V (2017). Non-specific chronic low back pain: differences in spinal kinematics in subgroups during functional tasks. Eur Spine J.

[22] Pourahmadi, Takamjani IE, Jaberzadeh S (2018). Kinematics of the spine during sit-to-stand movement using motion analysis systems: a systematic review of literature. J Sport Rehabil.

[23] Staartjes VE, Schroeder ML (2018). The five-repetition sit-to-stand test: evaluation of a simple and objective tool for the assessment of degenerative pathologies of the lumbar spine. J Neurosurg Spine.

[24] Actis JA, Nolasco LA, Gates DH, Silverman AK (2018). Lumbar loads and trunk kinematics in people with a transtibial amputation during sit-to-stand. J Biomech.

[25] Riley PO, Krebs DE, Popat RA (1997). Biomechanical analysis of failed sit-to-stand. IEEE Trans Rehabil Eng.

[26] Liu F-Y, Wang T, Yang S-D (2016). Incidence and risk factors for proximal junctional kyphosis: a meta-analysis. Eur Spine J.

[27] Hyun S-J, Kim YJ, Rhim S-C (2016). Patients with proximal junctional kyphosis after stopping at thoracolumbar junction have lower muscularity, fatty degeneration at the thoracolumbar area. Spine J.

[28] Yagi M, King AB, Boachie-Adjei O (2012). Incidence, risk factors, and natural course of proximal junctional kyphosis surgical outcomes review of adult idiopathic scoliosis. Minimum 5 years of follow-up. Spine.

[29] Ignasiak D, Rüeger A, Sperr R, Ferguson SJ (2018). Thoracolumbar spine loading associated with kinematics of the young and the elderly during activities of daily living. J Biomech.

