# Supplementary material for: ISSLS PRIZE IN BIOENGINEERING SCIENCE 2019: biomechanical changes in dynamic sagittal balance and lower limb compensatory strategies following realignment surgery in adult spinal deformity patients
Source: Eur Spine J. 2019 Mar 2;28(5):905–13. doi: 10.1007/s00586-019-05925-2 (PMC6536471; doi:10.1007/s00586-019-05925-2)
Supplement: Supplementary file 1 — Supplementary material 1 (PPTX 1132 kb) [file 586_2019_5925_MOESM1_ESM.pptx]

## Slide 1
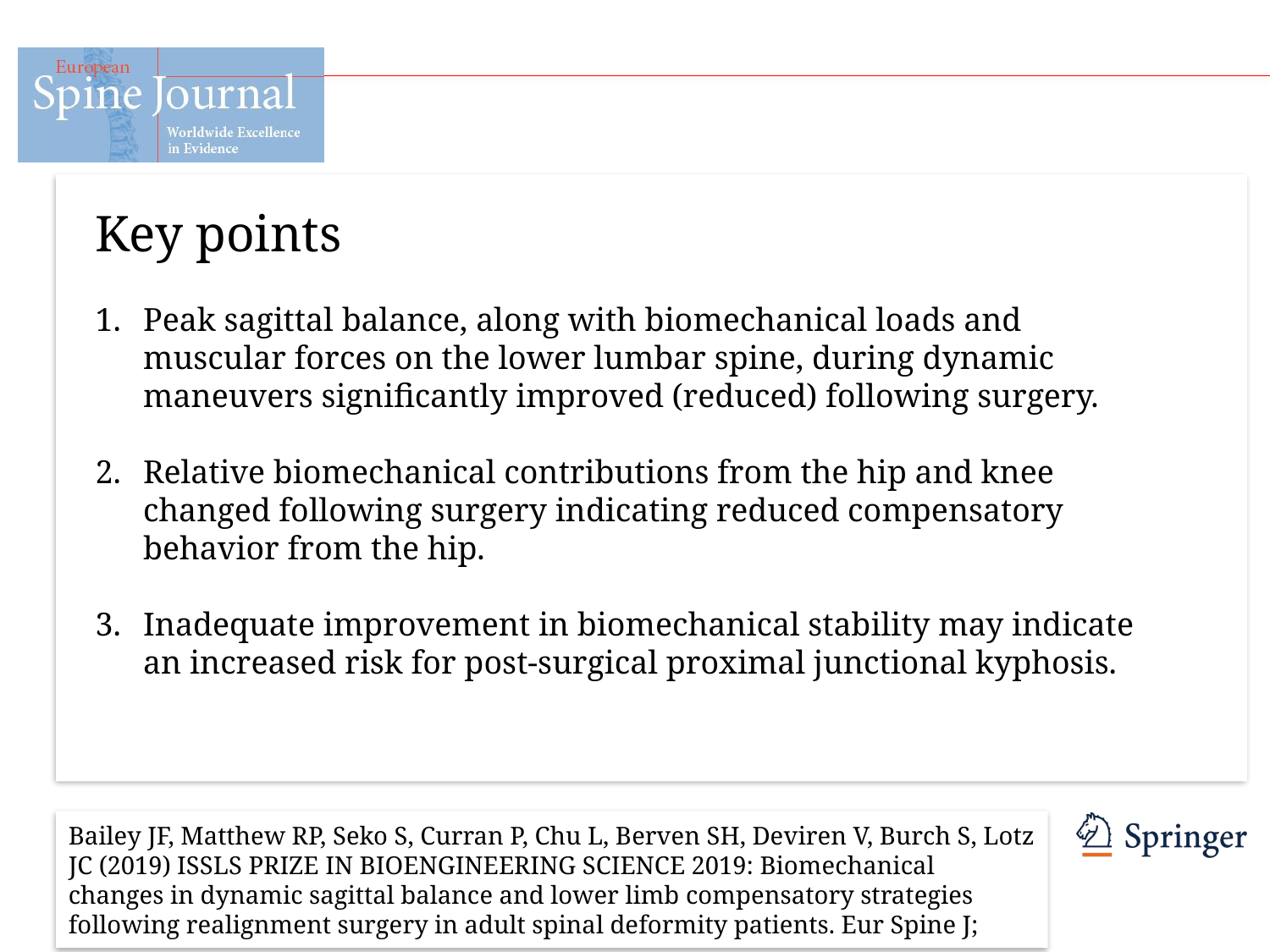

Key points
Peak sagittal balance, along with biomechanical loads and muscular forces on the lower lumbar spine, during dynamic maneuvers significantly improved (reduced) following surgery.
Relative biomechanical contributions from the hip and knee changed following surgery indicating reduced compensatory behavior from the hip.
Inadequate improvement in biomechanical stability may indicate an increased risk for post-surgical proximal junctional kyphosis.
Bailey JF, Matthew RP, Seko S, Curran P, Chu L, Berven SH, Deviren V, Burch S, Lotz JC (2019) ISSLS PRIZE IN BIOENGINEERING SCIENCE 2019: Biomechanical changes in dynamic sagittal balance and lower limb compensatory strategies following realignment surgery in adult spinal deformity patients. Eur Spine J;

## Slide 2
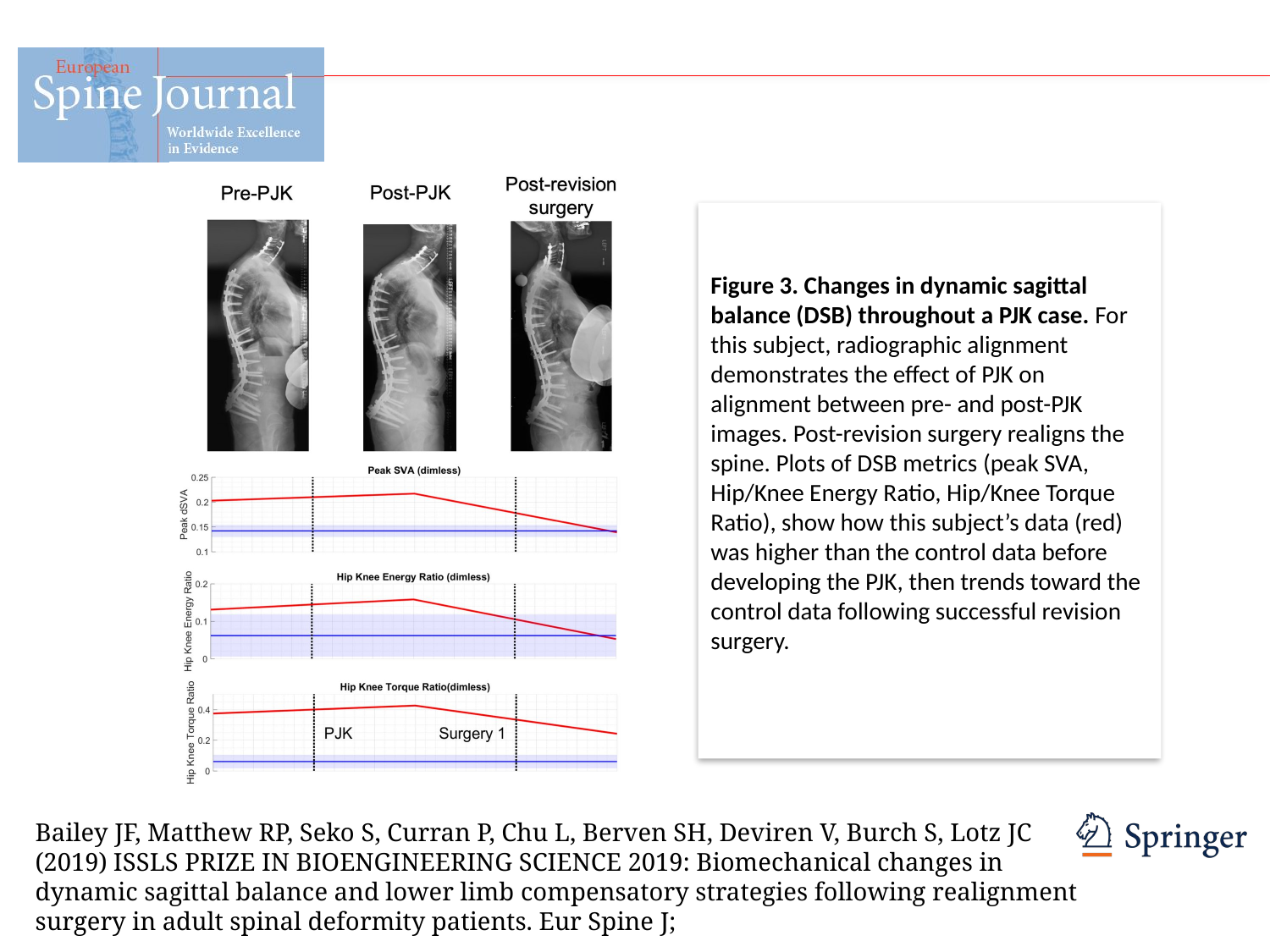

Figure 3. Changes in dynamic sagittal balance (DSB) throughout a PJK case. For this subject, radiographic alignment demonstrates the effect of PJK on alignment between pre- and post-PJK images. Post-revision surgery realigns the spine. Plots of DSB metrics (peak SVA, Hip/Knee Energy Ratio, Hip/Knee Torque Ratio), show how this subject’s data (red) was higher than the control data before developing the PJK, then trends toward the control data following successful revision surgery.
Bailey JF, Matthew RP, Seko S, Curran P, Chu L, Berven SH, Deviren V, Burch S, Lotz JC
(2019) ISSLS PRIZE IN BIOENGINEERING SCIENCE 2019: Biomechanical changes in
dynamic sagittal balance and lower limb compensatory strategies following realignment
surgery in adult spinal deformity patients. Eur Spine J;

## Slide 3
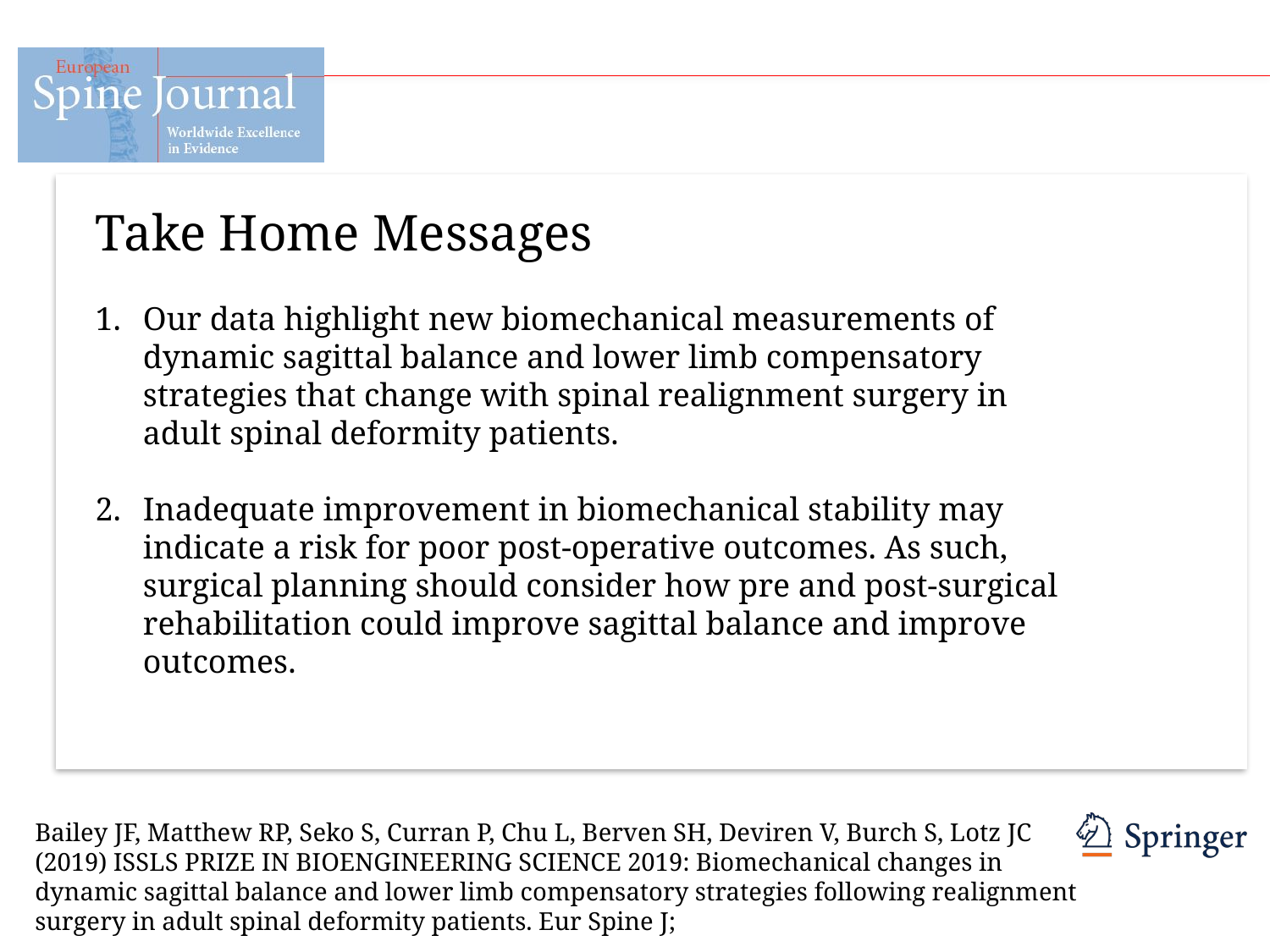

Take Home Messages
Our data highlight new biomechanical measurements of dynamic sagittal balance and lower limb compensatory strategies that change with spinal realignment surgery in adult spinal deformity patients.
Inadequate improvement in biomechanical stability may indicate a risk for poor post-operative outcomes. As such, surgical planning should consider how pre and post-surgical rehabilitation could improve sagittal balance and improve outcomes.
Bailey JF, Matthew RP, Seko S, Curran P, Chu L, Berven SH, Deviren V, Burch S, Lotz JC
(2019) ISSLS PRIZE IN BIOENGINEERING SCIENCE 2019: Biomechanical changes in
dynamic sagittal balance and lower limb compensatory strategies following realignment
surgery in adult spinal deformity patients. Eur Spine J;
